# Risk of major adverse cardiovascular events in subjects with asymptomatic mild carotid artery stenosis

**DOI:** 10.1038/s41598-018-23125-8

**Published:** 2018-03-16

**Authors:** Hyunwook Kwon, Hong-Kyu Kim, Sun U. Kwon, Seung-Whan Lee, Min-Ju Kim, Jee Won Park, Minsu Noh, Youngjin Han, Tae-Won Kwon, Yong-Pil Cho

**Affiliations:** 1 Department of Surgery, University of Ulsan College of Medicine, Asan Medical Center, Seoul, Republic of Korea; 2 Department of Health Screening and Promotion Center, University of Ulsan College of Medicine, Asan Medical Center, Seoul, Republic of Korea; 3 Department of Neurology, University of Ulsan College of Medicine, Asan Medical Center, Seoul, Republic of Korea; 4 Department of Internal Medicine, University of Ulsan College of Medicine, Asan Medical Center, Seoul, Republic of Korea; 5 Department of Clinical Epidemiology and Biostatistics, University of Ulsan College of Medicine, Asan Medical Center, Seoul, Republic of Korea

## Abstract

This study aimed to test the hypothesis that the risk of major adverse cardiovascular events (MACE) is similar for subjects with asymptomatic mild and moderate carotid artery stenosis (CAS). We enrolled a total of 453 subjects with asymptomatic CAS (30–69%) detected on baseline screening Doppler ultrasound (DUS) examination between January 2008 and December 2010. The follow-up DUS findings and MACE occurrence (fatal or nonfatal myocardial infarction or stroke and all-cause mortality) were compared between subjects with mild (30–49%) and moderate (50–69%) CAS during the 8-year follow-up period. There was no significant difference in the occurrence of MACE between subjects with mild (n = 289) and moderate (n = 164) CAS (13.8% vs. 15.9%, respectively; *p* = 0.56), although there was a nonsignificant trend toward an increased risk of major ipsilateral stroke in subjects with moderate CAS (1.4% vs. 4.3%; *p* = 0.06). Multivariate regression analysis indicated that worsening CAS was independently associated with MACE occurrence (hazard ratio [HR], 4.40; 95% confidence interval [CI], 2.65–7.27; *p* < 0.01), whereas an increased serum high-density lipoprotein cholesterol level was correlated with a decreased risk of MACE (HR, 0.42; 95% CI, 0.23–0.75; *p* < 0.01). The cumulative risk of MACE in subjects with asymptomatic mild CAS is similar to that in subjects with asymptomatic moderate CAS.

## Introduction

Cardiovascular disease (CVD) is the leading cause of morbidity and mortality worldwide, and its prevention is less costly than the treatment of its complications. Hence, the identification of subclinical disease during the asymptomatic phase is an important public health and economic goal^1^. Although cardiovascular risk assessment based on conventional risk factors is generally recommended, a significant percentage of persons—irrespective of conventional risk factors—present with subclinical atherosclerosis on Doppler ultrasound (DUS) imaging^1–5^. Therefore, carotid DUS screening could be a valuable tool to assist in CVD risk stratification for the application of preventive strategies in persons with few conventional risk factors. However, the 2013 American College of Cardiology/American Heart Association guidelines recommend that it is reasonable to repeat DUS screening annually for persons with moderate carotid artery stenosis (CAS) but not for those with mild CAS^2,6–8^, and limited studies to date have examined the natural course of asymptomatic mild CAS.

Owing to recent advances in medical treatment, the annual risk of stroke has decreased to <1.5% in persons with moderate to severe CAS receiving the best medical treatment^9,10^, and the difference in the risk for major adverse cardiovascular events (MACE) between persons with mild and moderate CAS could also be decreased. Therefore, this study aimed to test the hypothesis that the risk of MACE is similar between subjects with asymptomatic mild CAS and those with asymptomatic moderate CAS, as determined by DUS screening, in the era of cutting-edge medical treatment.

## Results

### Study population

A total of 453 subjects without clinical CVD at baseline and with a reported CAS in the range of 30–69% on baseline DUS for health screening and regular follow-ups were included in the analysis. Eligible subjects were stratified into two groups according to the degree of CAS as follows: mild CAS (n = 289, 63.8%) and moderate CAS (n = 164, 36.2%). The baseline characteristics of the subjects are presented in Table 1. Subjects with moderate CAS were less often obese (*p* = 0.01) and had a higher prevalence of chronic kidney disease (*p* = 0.03) than those with mild CAS. Total cholesterol (*p* = 0.03) and low-density lipoprotein cholesterol (*p = *0.01) levels were significantly lower in subjects with moderate CAS. The proportion of subjects taking antiplatelet medications (*p* = 0.05) was significantly higher in those with moderate CAS. The baseline characteristics of the subjects stratified according to antiplatelet medication are presented in Supplementary Table 1; subjects who used antiplatelet medication had more atherosclerosis risk factors than those who did not take antiplatelet medication. Although MACE occurrence (10.8% vs. 18.0%, *p* = 0.08) showed no significant difference between subjects with and without antiplatelet medication in the mild CAS group, the subjects with antiplatelet medication in the moderate CAS group showed higher MACE incidence than those without antiplatelet medication (8.9% vs. 22.4%, *p* = 0.02).

**Table 1:** Baseline characteristics of the study population stratified according to CAS degree. Continuous data are presented as means ± standard deviations, whereas categorical data are presented as numbers (%). CAS, carotid artery stenosis; HDL, high-density lipoprotein; LDL, low-density lipoprotein.

	Total	Mild CAS	Moderate CAS	*p*-value
Number of patients	453 (100)	289 (63.8)	164 (36.2)	
Mean age (years)	64.8 ± 8.0	64.6 ± 7.6	65.3 ± 8.5	0.34
Male sex	361 (79.7)	236 (81.7)	125 (76.2)	0.17
Body mass index (kg/m^2^)	23.5 ± 2.7	23.7 ± 2.6	23.1 ± 2.9	0.01
**Risk factor**				
Diabetes mellitus	125 (27.6)	80 (27.7)	45 (27.4)	0.96
Hypertension	198 (43.7)	129 (44.6)	69 (42.1)	0.60
Smoking	114 (25.2)	72 (24.9)	42 (25.6)	0.87
Chronic kidney disease	45 (9.9)	22 (7.6)	23 (14.0)	0.03
Dyslipidemia	288 (63.6)	190 (65.7)	98 (59.8)	0.20
Atrial fibrillation	11 (2.4)	7 (2.4)	4 (2.4)	0.99
**Medication**				
Antiplatelet use	207 (45.7)	122 (42.2)	85 (51.8)	0.05
Statin use	141 (31.1)	91 (31.5)	50 (30.5)	0.83
Anticoagulation	8 (1.8)	3 (1.0)	5 (3.0)	0.12
**Lipid profile** (**mg/dL**)				
Total cholesterol	176.2 ± 36.6	179.0 ± 36.3	171.3 ± 36.7	0.03
Triglyceride	128.2 ± 71.7	128.5 ± 61.7	127.8 ± 86.7	0.91
LDL-cholesterol	107.7 ± 32.7	110.6 ± 33.0	102.5 ± 31.6	0.01
HDL-cholesterol	51.4 ± 12.4	51.8 ± 12.5	50.7 ± 12.0	0.36

### Association between degree of CAS and MACE

During the mean follow-up period of 4.7 ± 2.2 years (maximum follow-up, 8 years), the MACE incidence was 13.8% in subjects with mild CAS and 15.9% in those with moderate CAS (Table 2). The difference was not significant between subjects with mild and moderate CAS (*p* = 0.56). The risk of major ipsilateral stroke (1.4% vs. 4.3%, *p* = 0.06) showed a numerically increasing trend in subjects with moderate CAS; however, no significant difference was noted in the incidence of any stroke (10.0% vs. 11.0%, *p = *0.75), major stroke (2.8% vs. 4.9%, *p* = 0.24), myocardial infarction (MI) (2.4% vs. 3.7%, *p = *0.45) or all-cause mortality (1.4% vs. 1.2%, *p = *0.88) between subjects with mild and moderate CAS. According to the degree of CAS (mild vs. moderate) and stroke causes, there were 7 cardioembolic (2.4%), 9 lacunar (3.1%), and 13 large-artery (4.5%) strokes in subjects with mild CAS, whereas there were 6 cardioembolic (3.7%), 3 lacunar (1.8%), and 9 large-artery (5.5%) strokes in subjects with moderate CAS (Table 3).

**Table 2:** MACE in the study subjects stratified according to CAS degree. Values are presented as numbers of subjects (%). CAS, carotid artery stenosis; MACE, major adverse cardiovascular events.

	Total	Mild CAS	Moderate CAS	*p*-value
MACE*	66 (14.6)	40 (13.8)	26 (15.9)	0.56
Any stroke	47 (10.4)	29 (10.0)	18 (11.0)	0.75
Major stroke	16 (3.5)	8 (2.8)	8 (4.9)	0.24
Major ipsilateral stroke	11 (2.4)	4 (1.4)	7 (4.3)	0.06
Minor stroke	31 (6.8)	21 (7.3)	10 (6.1)	0.64
Minor ipsilateral stroke	21 (4.6)	14 (4.8)	7 (4.3)	0.78
Myocardial infarction	13 (2.9)	7 (2.4)	6 (3.7)	0.45
All-cause mortality	6 (1.3)	4 (1.4)	2 (1.2)	0.88

*MACE occurrence during the follow-up period.

**Table 3:** Causes of strokes in the study subjects stratified according to CAS degree. Values are presented as numbers of subjects (%). CAS, carotid artery stenosis.

	Mild CAS (n = 289)			Moderate CAS (n = 164)		
	Total	Minor	Major	Total	Minor	Major
Cardioembolic	7 (2.4)	3 (1.0)	4 (1.4)	6 (3.7)	2 (1.2)	4 (2.4)
Lacunar	9 (3.1)	7 (2.4)	2 (0.7)	3 (1.8)	3 (1.8)	0 (0.0)
Large-artery	13 (4.5)	11 (3.8)	2 (0.7)	9 (5.5)	5 (3.0)	4 (2.4)

**Figure 1:**
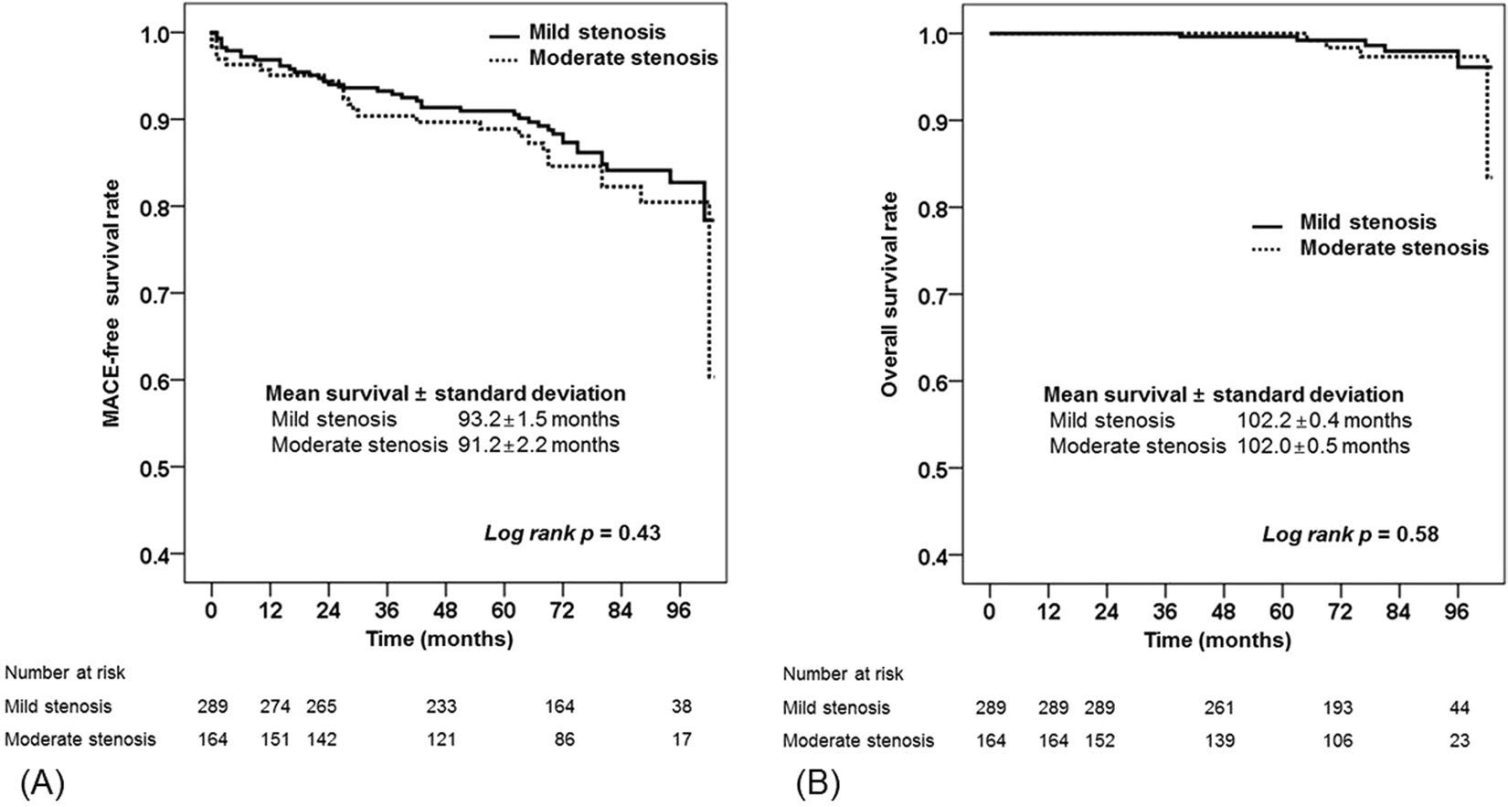
Kaplan–Meier analyses of cumulative event-free rates. (**A**) MACE-free and (**B**) overall survival rates of subjects with mild and moderate carotid artery stenosis. MACE, major adverse cardiovascular events.

**Figure 2:**
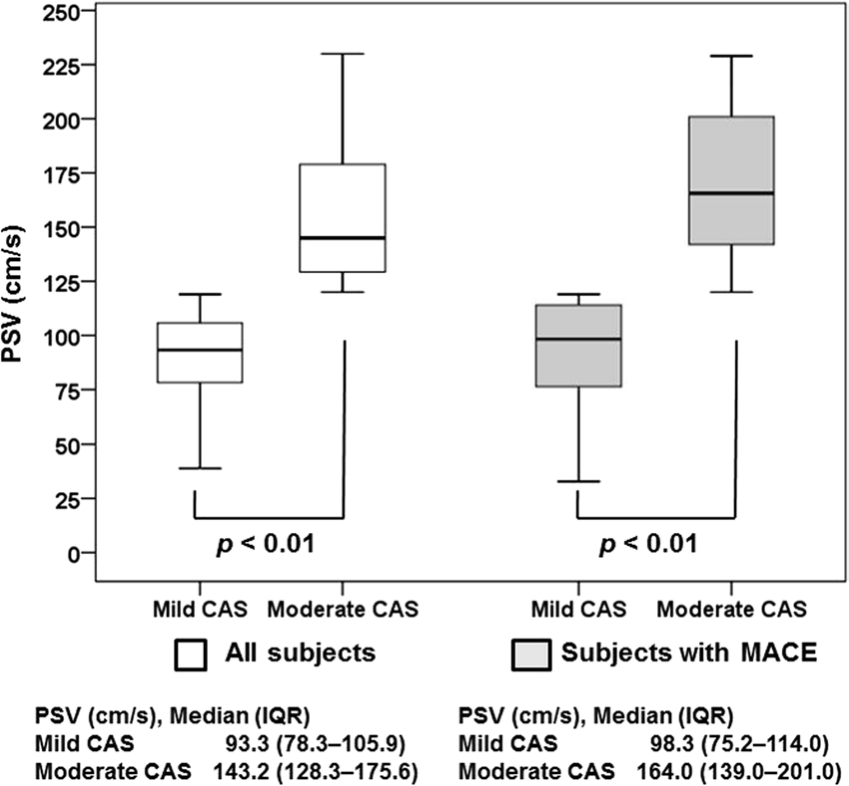
Boxplot of peak systolic velocity (PSV) on baseline Doppler ultrasound imaging. The distribution of PSV values in all subjects included in this study and in subjects with MACE occurrence during the follow-up period revealed a significant difference between mild and moderate CAS. CAS, carotid artery stenosis; IQR, interquartile range; MACE, major adverse cardiovascular events.

Kaplan-Meier analysis revealed no significant differences in the MACE-free (*p* = 0.43) and overall (*p* = 0.58) survival rates between subjects with mild and moderate CAS (Fig. 1). To test whether medians of the compared groups (mild CAS vs. moderate CAS) were different, the peak systolic velocity values were analyzed with the Mann-Whitney *U* test. The distribution of peak systolic velocity values in all enrolled subjects (mild CAS vs. moderate CAS; median [IQR], 93.3 cm/s [78.3–105.9 cm/s] vs. 143.2 cm/s [128.3–175.6 cm/s]; *p* < 0.01) and in subjects with MACE occurrence during the follow-up period (mild CAS vs. moderate CAS; median [IQR], 98.3 cm/s [75.2–114.0 cm/s] vs. 164.0 cm/s [139.0–201.0 cm/s]; *p* < 0.01) revealed a significant difference between mild and moderate CAS (Fig. 2).

### Analysis of clinical variables associated with MACE

Clinical variables associated with MACE occurrence were analyzed using univariate and multivariate Cox proportional hazards regression analysis (Table 4). Age, diabetes mellitus, high-density lipoprotein cholesterol level, use of antiplatelet medication, and CAS progression were significantly associated with MACE occurrence in the univariate analyses. After adjustment for confounding variables, multivariate Cox proportional hazard regression analysis indicated that high-density lipoprotein cholesterol level had a protective effect on MACE occurrence (HR, 0.42; 95% CI, 0.23–0.75; *p* < 0.01), whereas CAS progression during the follow-up period increased the risk of MACE 4.40-fold (95% CI, 2.65–7.27; *p* < 0.01). Although diabetes mellitus (HR, 1.60; 95% CI, 0.98–2.64; *p* = 0.06) showed trends associated with an increased risk of MACE occurrence, this was not statistically significant. Multivariate analyses of the association between clinical variables and CAS progression indicated that older age (HR, 1.04; 95% CI, 1.01–1.06; *p* < 0.01), smoking (HR, 1.84; 95% CI, 1.24–2.73; *p* < 0.01), and moderate CAS (HR, 1.59; 95% CI, 1.10–2.30; *p* = 0.01) were independently associated with CAS progression during the follow-up period (Table 5).

**Table 4:** Factors associated with the occurrence of major adverse cardiovascular events. CAS, carotid artery stenosis; CI, confidence interval; HDL, high-density lipoprotein; HR, hazard ratio; LDL, low-density lipoprotein; NA, not applicable.

	Univariate analysis		Multivariate analysis	
	HR (95% CI)	*p*-value	HR (95% CI)	*p*-value
Age (years)	1.04 (1.01–1.07)	0.01	1.02 (0.99–1.06)	0.16
Male sex	1.11 (0.60–2.08)	0.74	NA	NA
Body mass index (kg/m^2^)	1.03 (0.95–1.12)	0.49	NA	NA
Diabetes mellitus	1.86 (1.14–3.03)	0.01	1.60 (0.98–2.64)	0.06
Hypertension	1.47 (0.91–2.39)	0.12	NA	NA
Smoking	1.19 (0.69–2.04)	0.54	NA	NA
Chronic kidney disease	1.52 (0.75–3.08)	0.24	NA	NA
Dyslipidemia	1.43 (0.84–2.42)	0.19	NA	NA
Total cholesterol ≥220 mg/dL	0.92 (0.44–1.92)	0.82	NA	NA
Triglycerides ≥150 mg/dL	0.96 (0.55–1.67)	0.89	NA	NA
LDL-cholesterol ≥140 mg/dL	1.03 (0.53–2.02)	0.93	NA	NA
HDL-cholesterol ≥40 mg/dL	0.50 (0.28–0.88)	0.01	0.42 (0.23–0.75)	<0.01
Antiplatelet use	1.99 (1.21–3.28)	0.01	1.60 (0.96–2.66)	0.07
Statin use	1.12 (0.68–1.87)	0.65	NA	NA
CAS progression	4.44 (2.72–7.24)	<0.01	4.40 (2.65–7.27)	<0.01
CAS				
Mild CAS	Reference			
Moderate CAS	1.22 (0.74–2.00)	0.43	NA	NA

**Table 5:** Factors associated with risk of CAS progression. CAS, carotid artery stenosis; CI, confidence interval; HDL, high-density lipoprotein; HR, hazard ratio; LDL, low-density lipoprotein; NA, not applicable.

	Univariate analysis		Multivariate analysis	
	HR (95% CI)	*p*-value	HR (95% CI)	*p*-value
Age (years)	1.04 (1.01−1.06)	<0.01	1.04 (1.01−1.06)	<0.01
Male sex	1.17 (0.72−1.92)	0.53	NA	NA
Body mass index (kg/m^2^)	0.98 (0.91−1.05)	0.51	NA	NA
Diabetes mellitus	1.19 (0.80−1.76)	0.39	NA	NA
Hypertension	1.13 (0.78−1.63)	0.52	NA	NA
Smoking	1.64 (1.11−2.42)	0.01	1.84 (1.24−2.73)	<0.01
Chronic kidney disease	1.62 (0.96−2.75)	0.07	1.33 (0.77–2.29)	0.31
Dyslipidemia	1.03 (0.71−1.51)	0.87	NA	NA
Total cholesterol ≥220 mg/dL	1.09 (0.65−1.86)	0.74	NA	NA
Triglyceride ≥150 mg/dL	0.91 (0.60−1.37)	0.63	NA	NA
LDL-cholesterol ≥140 mg/dL	1.09 (0.66−1.81)	0.73	NA	NA
HDL-cholesterol ≥40 mg/dL	0.72 (0.41−1.26)	0.25	NA	NA
Antiplatelet use	1.22 (0.85−1.76)	0.29	NA	NA
Statin use	1.08 (0.74−1.59)	0.69	NA	NA
CAS				
Mild CAS	Reference			
Moderate CAS	1.66 (1.15−2.40)	<0.01	1.59 (1.10−2.30)	0.01

## Discussion

This study tested and proved the hypothesis that the cumulative risk of MACE is similar between subjects with asymptomatic mild and moderate CAS. In our analysis, stroke was the most common MACE in subjects with asymptomatic mild and moderate CAS during the follow-up period, and we attribute this result to the current advanced medical treatments and the decreased risk of stroke in subjects with asymptomatic moderate CAS receiving optimal medical treatment. This finding highlights the fact that carotid atherosclerosis is a dynamic process associated with the risk of MACE occurrence, which persists over time even in cases of asymptomatic mild CAS^11^. Thus, further studies are needed to determine new guidelines for the screening of subclinical CVD and the treatment of its complications.

Although cardiovascular risk assessment based on conventional risk factors is generally recommended, its predictive ability is only moderate^1,5^. Many population-based studies assessing the association between carotid intima-media thickness and MACE have shown that carotid intima-media thickness is a strong predictor of MACE occurrence^7,12^. However, intima-media thickness mainly due to aging or hypertension does not necessarily reflect the dynamic process of atherosclerosis^13^. Several studies have suggested that other methods, such as measurements of plaque area and volume, may predict the risk of MACE better than CAS measurement. However, while CAS measurements are largely based on analysis of the peak systolic velocity and end-diastolic velocity^6,14–16^, these other imaging methodologies are more complex, time-consuming, expensive, and not readily available^17,18^. For routine screening of subclinical atherosclerosis of the carotid artery in the general population, availability, ease of measurement, and cost are essential when choosing the imaging methodology^17^. Imaging of carotid plaque and measurement of CAS degree, based on the analysis of the peak systolic velocity and end-diastolic velocity, is a simple, noninvasive, and cost-effective method, and its reported overall accuracy is up to 92%^19^. The CAS degree determined with DUS in subjects without clinical CVD has been reported to be an independent predictor for MACE occurrence^4,20^. Therefore, DUS screening to evaluate the CAS degree in the general population could be a valuable tool that can assist in cardiovascular risk stratification for the application of preventive strategies.

In our study, the average annual rate of MACE in subjects with asymptomatic moderate CAS was 3.4%, and there was no significant difference compared with that in the SMART study^4^. The annual rate of major ipsilateral stroke in subjects with moderate CAS was 0.9%, which was relatively lower than that in subjects with moderate CAS in early randomized clinical trials of carotid endarterectomy (NASCET and European Carotid Surgery Trial) but similar to the recent large cohort studies that reported an annual rate of stroke <1.5%^4,10,21–23^. In our study, the annual risk of major ipsilateral stroke had a slightly increased trend in subjects with asymptomatic moderate CAS compared to those with asymptomatic mild CAS, whereas the risk of any stroke was similar between these subjects. Although the CAS degree has been reported to be a significant predictor of future MI and its severity^1,4,6^, no significant difference was noted in the incidence of MI between subjects with mild and moderate CAS in our study. According to data from the Department of Measurement and Health Information of the World Health Organization, there is a significant difference in the risk of MACE between different racial/ethnic groups; stroke is more prominent than MI in the general Asian population compared with the general Western population^11,24–26^. We speculated that the incidence of MI was not high enough to indicate a difference between subjects with asymptomatic mild and moderate CAS because our study cohort consisted of only Korean Asians. Alternatively, mild and moderate CAS may be comparable risk factors of subclinical MI in the Asian population. Although previous studies have shown that the risk of MACE is related to the presence and severity of CAS^17^, there are very few reports available for Asians, and the association between asymptomatic mild CAS and MACE occurrence remains controversial^27,28^. The present study evaluated the impact of asymptomatic mild CAS on the subsequent occurrence of MACE in an Asian population and found that asymptomatic mild CAS and asymptomatic moderate CAS were comparable risk factors of subsequent MACE occurrence. Our findings may serve as novel data that could aid in the optimal surveillance and management of risk of MACE in Asian population without clinical CVD but with mild CAS detected on DUS screening.

On multivariate analyses, our data showed that serum high-density lipoprotein cholesterol levels and CAS progression were significantly associated with MACE occurrence, whereas older age, smoking, and moderate CAS were significant risk factors for CAS progression. CAS progression is known to be a potential marker of ischemic neurologic outcomes^29–31^. Hirt reported that fast progression of CAS was associated with an increased incidence of ipsilateral neurologic events, although carotid luminal narrowing itself was not a significant variable for ipsilateral events^30^. Bertges *et al*. suggested that follow-up of asymptomatic subjects with serial DUS surveillance would be beneficial by demonstrating that CAS progression was a better predictor of ischemic stroke than a single measurement of CAS^31^. Our data indicated that an increased serum high-density lipoprotein cholesterol level was significantly associated with a decreased risk of MACE in subjects without clinical CVD, whereas CAS progression was significantly associated with an increased risk of MACE. Although some controversy exists about the optimal level of serum high-density lipoprotein cholesterol, serum high-density lipoprotein cholesterol is believed to protect against MACE by promoting reverse cholesterol transport and serving as a predictor of atherosclerotic burden^32,33^. Our study cohort consisted of preselected healthy subjects without clinical CVD. Antiplatelet medications, prescribed by the subjects’ health-care providers according to their individual atherosclerosis risk factors, were used only for subjects with atherosclerosis risk factors. Although subjects using antiplatelet medications seemed to show a trend toward an increased risk of MACE occurrence in our analysis, this was not because the antiplatelet medication itself increased the MACE occurrence but because subjects who used antiplatelet medication had more atherosclerosis risk factors than those who did not take antiplatelet medication. Antiplatelet medication did not affect the CAS progression in Cox regression analysis.

The present study had certain limitations. First, owing to its retrospective design, this study may have been subject to selection and information biases. Hence, the incidence of MACE may have been overestimated, and the number of excluded patients was considerable. Second, our study cohort comprised only subjects of Asian descent; thus, because there may be racial/ethnic differences in the prevalence of MACE, our findings should be interpreted with caution with respect to different racial and ethnic groups. Third, there could be inter-observer errors affecting our analyses because the reliability of DUS examination depends highly on the expertise of the examiner and the knowledge and experience of the interpreting physician. To correct these errors, all baseline and follow-up tests were performed by accredited radiologists, and all images were reviewed by experienced vascular surgeons who were unaware of the subjects’ general health status. To ensure that the distribution of the CAS degree around the border was not the reason why the clinical outcomes between subjects with mild and moderate CAS were similar, we showed that medians and interquartile ranges of the compared groups were significantly different in all subjects included in this study and in subjects with MACE occurrence during the follow-up period. Finally, based on the relatively small sample size of the study cohort, this study was likely underpowered to prove the hypothesis that the risk of MACE is similar for subjects with asymptomatic mild and moderate CAS.

## Conclusion

In conclusion, subjects with asymptomatic mild and moderate CAS had similar risks of MACE. Considering that the risk of MACE in healthy subjects with asymptomatic moderate to severe CAS has certainly decreased owing to improved medical treatment, randomized controlled trials for new guidelines on DUS screening of subclinical CVD and preventive treatment of its complications in the general population are currently needed. Our results could provide valuable background evidence for further large cohort studies.

## Methods

### Study design and population

In this single-center, retrospective, observational study, we analyzed data extracted from subjects’ medical records. The study protocol was approved by the institutional review board of the Asan Medical Center (2016–0900), which waived the need for informed consent because of its retrospective nature. All methods were performed in accordance with the relevant guidelines and regulations. We recruited subjects from among those aged >50 years who visited the Health Screening and Promotion Center of our hospital between January 2008 and December 2010 and underwent baseline cardiovascular screening including cardiac enzyme levels, 12-lead electrocardiogram, and routine carotid DUS examination. The exclusion criteria were evidence of CVD including cerebral infarction, MI, angina, heart failure, and valvular heart disease; history of carotid or coronary revascularization; and CAS in the range of 0–30% diameter reduction or severe CAS (≥70% stenosis based on the North American Symptomatic Carotid Endarterectomy Trial [NASCET] criteria)^6^ on baseline DUS. Subjects who were lost to follow-up or did not repeat follow-up DUS within at least 1 year were also excluded from the analysis. Among these subjects, 453 Asians with asymptomatic CAS on baseline DUS, ranging from 30–69% based on the NASCET and velocity criteria (peak systolic velocity and end-diastolic velocity values)^21,34^, and normal cardiac enzyme levels and electrocardiogram were included in this study.

### Clinical data

DUS was performed by experienced radiologists in the Health Screening and Promotion Center of our hospital. The degree of CAS was estimated on the basis of peak systolic velocity and end-diastolic velocity values recorded from within the most stenotic segment in addition to plaque imaging (% diameter reduction stenosis)^11^. CAS was classified as either mild or moderate. Mild CAS was defined as 30–49% stenosis, determined through analysis of peak systolic velocity <125 cm/s and end-diastolic velocity <40 cm/s. Moderate CAS was defined as 50–69% stenosis, determined through analysis of the peak systolic velocity in the range of 125–230 cm/s and end-diastolic velocity in the range of 40–100 cm/s^11,34^. In the case of a discrepancy in the CAS degree determined according to luminal narrowing based on the NASCET and velocity criteria, the estimation of CAS was based primarily on the velocity criteria. The most recent DUS findings were used to determine worsening of CAS, which was defined as progression from mild to moderate stenosis or moderate to severe stenosis. In subjects with stenosis of both carotid arteries, the most severe stenotic artery was selected as the reference.

### Follow-up and outcomes of interest

All subjects included in this study visited the Health Screening and Promotion Center of our hospital at approximately 12-month intervals and underwent repeat carotid DUS. All medication adjustments were made by the subjects’ health-care provider at our hospital according to each individual’s atherosclerosis risk factors. Risk factors of interest, clinical characteristics, and long-term clinical outcomes for all subjects were recorded in an Excel database (Microsoft Corp., Redmond, WA, USA) and analyzed retrospectively.

The primary outcomes included the occurrence of MACE, defined as fatal or nonfatal stroke or MI, and all-cause mortality. Data on all MACE were centrally reviewed and adjudicated by an experienced neurologist or cardiologist. In the analysis, we included only ischemic strokes defined as previously^11,35^. Strokes were categorized as major or minor according to clinical severity^11^ and as cardioembolic, lacunar, or large-artery according to their underlying cause. MI was defined as previously^11^.

### Statistical analysis

Categorical variables are reported as frequencies or percentages and continuous variables as means and standard deviations. Categorical variables were compared using the chi-square test or Fisher’s exact test, as appropriate, whereas continuous variables were compared using the Student’s *t* test. Peak systolic velocity values, not distributed normally and presented as medians and interquartile ranges, were analyzed using the Mann–Whitney *U* test. The cumulative probability of events was estimated with Kaplan–Meier analysis and compared with that estimated with the log-rank test. Univariate and multivariate analyses of the association of clinical variables with each outcome and CAS progression were conducted with Cox proportional hazards modeling using the event of interest and period from study enrollment to the date of the event or last follow-up as the outcome. Univariate Cox proportional hazard regression models were fitted to calculate hazard ratios (HRs) with 95% confidence intervals (CIs) to estimate the association of clinical variables with the occurrence of MACE and CAS progression. Variables with a *p*-value of <0.1 on univariate analysis were included in multivariate Cox proportional hazard regression models, by using the backward elimination method. A *p*-value of <0.05 was considered statistically significant. Statistical analyses were performed with SPSS version 21.0 (SPSS Inc., Chicago, IL, USA).

### Data availability statement

The datasets generated and/or analyzed during the current study are available from the corresponding author on reasonable request.

## Electronic supplementary material

Supplementary Table 1
