# Supplementary material for: Risk of major adverse cardiovascular events in subjects with asymptomatic mild carotid artery stenosis
Source: Sci Rep. 2018 Mar 16;8:4700. doi: 10.1038/s41598-018-23125-8 (PMC5856768; doi:10.1038/s41598-018-23125-8)
Supplement: Supplementary file 1 — Supplementary Table 1 [file 41598_2018_23125_MOESM1_ESM.docx]

Original Research Article

**Risk of major adverse cardiovascular events in subjects with asymptomatic mild carotid artery stenosis**

Hyunwook Kwon^1^, Hong-Kyu Kim^2^, Sun U. Kwon^3^, Seung-Whan Lee^4^, Min-Ju Kim^5^, Jee Won Park^2^, Minsu Noh^1^, Youngjin Han^1^, Tae-Won Kwon^1^, Yong-Pil Cho^1*^

^1^Department of Surgery, University of Ulsan College of Medicine, Asan Medical Center, Seoul, Republic of Korea.

^2^Department of Health Screening and Promotion Center, University of Ulsan College of Medicine, Asan Medical Center, Seoul, Republic of Korea.

^3^Department of Neurology, University of Ulsan College of Medicine, Asan Medical Center, Seoul, Republic of Korea.

^4^Department of Internal Medicine, University of Ulsan College of Medicine, Asan Medical Center, Seoul, Republic of Korea.

^5^Department of Clinical Epidemiology and Biostatistics, University of Ulsan College of Medicine, Asan Medical Center, Seoul, Republic of Korea.

**^*^Corresponding Author:** Yong-Pil Cho, MD, Division of Vascular Surgery, Department of Surgery, University of Ulsan College of Medicine, Asan Medical Center, 88, Olympic-ro 43-gil, Songpa-gu, Seoul 05505, Republic of Korea

Phone: +82-2-3010-5039; Fax: +82-2-3010-6701; E-mail: [ypcho@amc.seoul.kr](mailto:ypcho@amc.seoul.kr)

**Running Head:** MACE in mild carotid artery stenosis

|  | Total | No antiplatelet | Antiplatelet | *p*-value |
| --- | --- | --- | --- | --- |
| Number of patients | 453 (100) | 246 (54.3) | 207(45.7) |  |
| Mean age (years) | 64.8 ± 8.0 | 63.9 ± 7.9 | 65.9 ± 7.9 | <0.01 |
| Male sex | 361 (79.9) | 190 (77.2) | 171 (82.6) | 0.16 |
| Body mass index (kg/m^2^) | 23.5 ± 2.7 | 23.6 ± 2.9 | 23.3 ± 2.4 | 0.18 |
| Risk factor |  |  |  |  |
| Diabetes mellitus | 125 (27.6) | 60 (24.4) | 65 (31.4) | 0.10 |
| Hypertension | 198 (43.7) | 98 (39.8) | 100 (48.3) | 0.07 |
| Smoking | 114 (25.2) | 64 (26.0) | 50 (24.2) | 0.65 |
| Chronic kidney disease | 45 (9.9) | 21 (8.5) | 24 (11.6) | 0.28 |
| Dyslipidemia | 288 (63.6) | 141 (57.3) | 147 (71.0) | <0.01 |
| Atrial fibrillation | 11 (2.4) | 2 (0.8) | 9 (4.3) | 0.02 |
| Lipid profile |  |  |  |  |
| Total cholesterol ≥ 220 mg/dL | 57 (12.6) | 32 (13.0) | 25 (12.1) | 0.77 |
| Triglycerides ≥ 150 mg/dL | 126 (27.8) | 71 (28.9) | 55 (26.6) | 0.59 |
| LDL-cholesterol ≥ 140 mg/dL | 67 (14.8) | 43 (17.5) | 24 (11.6) | 0.08 |
| HDL-cholesterol ≥ 40 mg/dL | 73 (16.1) | 29 (11.8) | 44 (21.3) | <0.01 |

**Supplementary Table 1**. Baseline characteristics of the study population stratified according to antiplatelet medication. Continuous data are presented as means ± standard deviations, whereas categorical data are presented as numbers (%). HDL, high-density lipoprotein; LDL, low-density lipoprotein.
